# Prediction of Incontinence after Robot-Assisted Radical Prostatectomy: Development and Validation of a 24-Month Incontinence Nomogram

**DOI:** 10.3390/cancers14071644

**Published:** 2022-03-24

**Authors:** Ruben M. Pinkhasov, Timothy Lee, Rogerio Huang, Bonnie Berkley, Alexandr M. Pinkhasov, Nicole Dodge, Matthew S. Loecher, Gaybrielle James, Elena Pop, Kristopher Attwood, James L. Mohler

**Affiliations:** 1 Department of Urology, Roswell Park Comprehensive Cancer Center, Buffalo, NY 14263, USA; dr.pinkhasov@gmail.com (R.M.P.); timothy_lee@urmc.rochester.edu (T.L.); rogerioh@buffalo.edu (R.H.); bberkley@sahrc.org (B.B.); pinkhasa@upstate.edu (A.M.P.); dodge.nicole1@mayo.edu (N.D.); matthew.loecher@tuhs.temple.edu (M.S.L.); gaybrielle.james@roswellpark.org (G.J.); med_leni@yahoo.com (E.P.); 2 Department of Biostatistics, Roswell Park Comprehensive Cancer Center, Buffalo, NY 14263, USA; kristopher.attwood@roswellpark.org

**Keywords:** robot-assisted radical prostatectomy, urinary incontinence, nomogram

## Abstract

**Simple Summary:**

Many men fear urinary leakage after radical surgery for prostate cancer and may even choose against operation for unrealistic fears of leakage. Many urologists are unaware of their own results, and some urologists who collect their results do so in different ways. We collected urinary leakage data from 680 men in a uniform and simple way at 6, 12, and 24 months after operation: no pads, 1–2 pads, or ≥3 pads required daily. We used many patient characteristics to identify the key factors that predict recovery of urinary control after operation: age, race, height and weight, and preoperative erectile function. Easy-to-use nomograms were constructed that should be tested by other urologists to make sure they perform equally well in their patients. Nomograms like these allow men and the urologists counseling them to share patient-specific information about the timeline for, and the chance of, recovery of urinary control after operation.

**Abstract:**

Incontinence after robot-assisted radical prostatectomy (RARP) is feared by most patients with prostate cancer. Many risk factors for incontinence after RARP are known, but a paucity of data integrates them. Prospectively acquired data from 680 men who underwent RARP January 2008–December 2015 and met inclusion/exclusion criteria were queried retrospectively and then divided into model development (80%) and validation (20%) cohorts. The UCLA-PCI-Short Form-v2 Urinary Function questionnaire was used to categorize perfect continence (0 pads), social continence (1–2 pads), or incontinence (≥3 pads). The observed incontinence rates were 26% at 6 months, 7% at 12 months, and 3% at 24 months. Logistic regression was used for model development, with variables identified using a backward selection process. Variables found predictive included age, race, body mass index, and preoperative erectile function. Internal validation and calibration were performed using standard bootstrap methodology. Calibration plots and receiver operating curves were used to evaluate model performance. The initial model had 6-, 12-, and 24-month areas under the curves (AUCs) of 0.64, 0.66, and 0.80, respectively. The recalibrated model had 6-, 12-, and 24-month AUCs of 0.52, 0.52, and 0.76, respectively. The final model was superior to any single clinical variable for predicting the risk of incontinence after RARP.

## 1. Introduction

Urinary continence after robot-assisted radical prostatectomy (RARP) is one of the main concerns for patients facing RARP. In fact, urinary continence may have a higher impact on quality of life than sexual function [1]. A rapid return to social or perfect continence after RARP is of utmost importance to some patients.

Despite all efforts, the incidence of post-prostatectomy incontinence varies from 2% to 66% in reported series depending on the definition of continence used, timing after the operation, and the type of surgical modality employed [2]. Walsh initially described continence as having no need for pads, but others have adopted a definition of zero or one safety pad for security in a day [3,4]. In a systematic review and meta-analysis of 51 surgical series between 2008 and 2011, the reported 12-month urinary incontinence rates were 4% to 31%, with mean of 16%, using the continence definition of zero pads [5]. Studies that used zero pads or one safety pad for security as the continence definition reported 12-month urinary incontinence rates that ranged from 8% to 11%, with a mean of 9% [5].

The most widely reported risk factors that predict continence recovery after RARP include age [6,7,8,9,10,11,12,13,14,15,16,17], body mass index (BMI) [6,15,17,18], severity of lower urinary tract symptoms (LUTS) [6,9,17], preoperative comorbidities [8,16], preoperative erectile function [6,9], prostate volume [19], urethral length [20,21], and surgeon experience [22,23]. We hypothesized that the more adverse risk factors for incontinence the patient has before RARP, the more likely he will remain incontinent at 2 years. The purpose of this study was to develop and validate a nomogram that predicts a patient’s probability of incontinence after RARP.

## 2. Materials and Methods

### 2.1. Patients and Procedures

**Figure 1:**
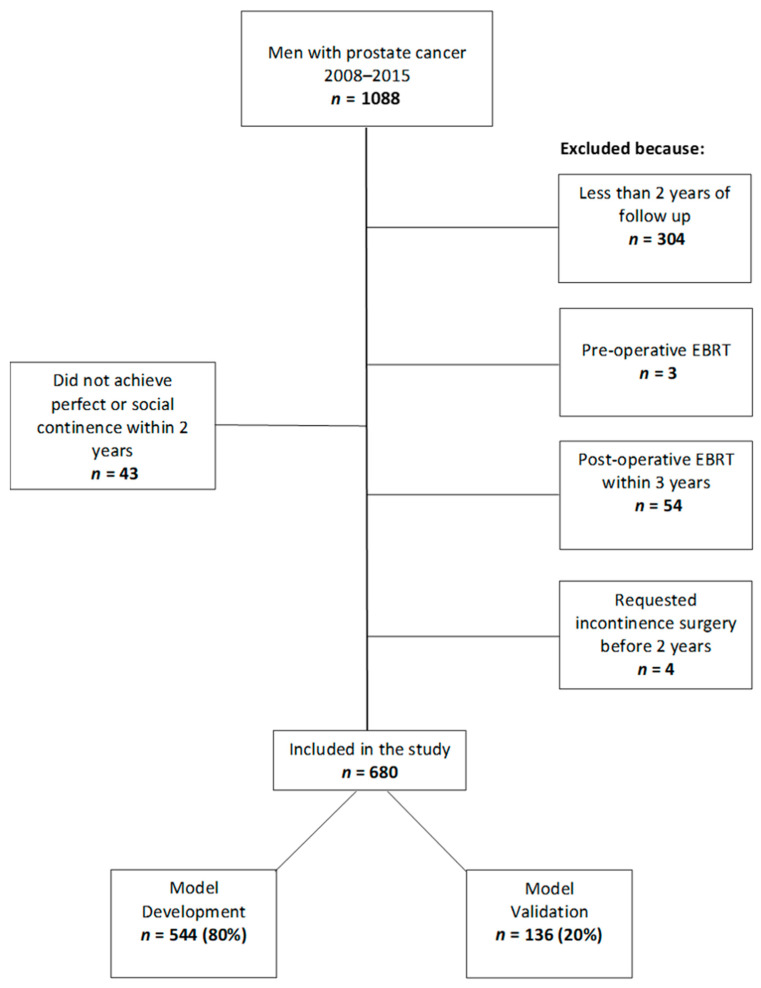
Flow diagram of patients excluded from the study and distribution of remaining patients into model development and validation cohorts; EBRT = external beam radiotherapy.

Institutional review board (IRB) approval was obtained to query retrospectively an institutional prospectively collected radical prostatectomy database. A total of 1088 patients were identified who underwent RARP by 5 different urologic surgeons from 5 January 2008 to 29 December 2014. Each urologic surgeon performed at least 100 RARPs before the initiation of the study and at least 25 RARPs per year during the study period. Study exclusion criteria were: (1) less than 2 years of follow-up; (2) preoperative external beam radiotherapy (EBRT); (3) post-operative EBRT within the first 3 years after RARP; (4) loss to follow up; (5) incomplete or missing UCLA Prostate Cancer Index Short Form version 2 (UCLA-PCI-SF v2) or International Prostate Symptom Score (IPSS) International Prostate Symptom Score (IPSS) preoperatively; and (6) preoperative incontinence requiring pad use. After applying the exclusion criteria, 680 patients were randomly assigned into two cohorts for model development (80%) and model validation (20%), and both were analyzed retrospectively (Figure 1). A uni- or bilateral nerve-sparing technique during RARP was performed when it was deemed not to increase significantly the risk of positive surgical margin. Pelvic lymphadenectomy was performed in higher-risk patients.

### 2.2. Continence Assessment and Follow up

Before RARP, patients completed the validated UCLA-PCI-SF v2 and IPSS questionnaires to assess baseline continence and lower urinary tract symptoms (LUTS), respectively. Foley catheter was removed 7–10 days after RARP. All patients were followed routinely with serum level of prostate-specific antigen (PSA), exam, and questionnaire (UCLA-PCI-SF v2) at 6 weeks after RARP every 6 months for 5 years, and annually thereafter unless prostate cancer was organ-confined and PSA was undetectable, in which case PSA, exam, and questionnaire were administered annually from years 1 to 5. Perfect continence was defined as 0 pads used, social continence was defined as 1 or 2 pads used, and incontinence was defined as ≥3 pads used daily. Patients who used 1 pad for security in 24 h were included in the social continence group.

### 2.3. Selection of Risk Factors for RARP Incontinence Nomogram

In over 60 surgical series that evaluated continence after radical prostatectomy from 1994 to 2016, predictors assessed for continence included age [6,7,8,9,10,11,12,13,14], BMI [6,18], severity of lower urinary tract symptoms (LUTS) [6,9,12], intra-operative estimated blood loss [12], Charlson comorbidity index [8,24], bladder neck invasion, pathologic stage [12,24], preoperative PSA [24], Gleason score, surgery date, margin status, prostate volume [19,24], type of surgery (bilateral nerve sparing, unilateral nerve sparing, non-nerve sparing, retropubic radical prostatectomy (RRP) vs laparoscopic radical prostatectomy vs RARP, hypothermic prostatectomy, extraperitoneal prostatectomy, Retzius sparing prostatectomy) [5,6,12,18,25,26], operative experience [11,23], urethral length [11,24], posterior repair [5], and preoperative erectile function [6,9].

The present study examined age, BMI, severity of LUTS (assessed using IPSS scores), presence of co-morbidity (measured using Charlson co-morbidity index), margins status, preoperative erectile function (assessed using UCLA-PCI-SF v2), and prostate volume (measured using prostatectomy pathology specimen) as predictors for continence and for the development of nomograms because these predictors have been studied in large cohorts, multiple studies have confirmed their predictive ability, and the predictors are generalizable.

### 2.4. Statistical Analysis

Patient characteristics were reported by cohort using mean, median, and standard deviation for continuous variables and frequency or relative frequencies for categorical variables. Time to perfect continence and social continence were summarized for the overall sample and by cohorts using standard Kaplan–Meier methods. Estimates of 6-, 12- and 24-month continence rates and median time to continence were obtained with 95% confidence intervals. Each patient had a unique number assigned and the Statistical Analysis System (SAS) was used to randomly assign the 680 patients into 2 cohorts: 544 (80%) for model development and 136 (20%) for model validation.

The initial prediction model for incontinence was developed using the model building cohort in several steps:

- First step: univariate associations between social continence (at 6-, 12-, and 24-months) and the preoperative variables in Table 1 were evaluated using logistic regression models. Odds ratios representing achieving at least social continence versus incontinence were obtained with 95% confidence intervals.
- Second step: main effects for a prediction model were selected using logistic regression and a bootstrap backward selection method (alpha exit = 0.1). The variables listed in Table 1 were included as candidate variables. The final main effects selected for both models were age, race, BMI, surgical margins status, and preoperative erectile function to maintain consistency among models.
- Third step: interactions for a prediction model were selected using logistic regression and a bootstrap forward selection method (alpha entry = 0.01), where all two-way interaction terms of the selected main effects were considered. No interaction terms were included in the models.
- Final step: a final logistic regression model was fit using the selected variables, and model coefficients were obtained using standard bootstrapping techniques. Odds ratios and corresponding 95% confidence intervals were obtained from model estimates.

The performance of the initial model was evaluated using calibration plots and receiver operating curves (ROC) with the corresponding area under the curves (AUC). The initial model was recalibrated using the calibration cohort and standard bootstrap methods. The final model was applied to the validation cohort and performance was assessed as described above. All analyses were completed in SAS v9.4 (Cary, NC, USA).

## 3. Results

Overall baseline patient characteristics were summarized (Table 1). Mean age at RARP was 61 (SD ± 6) years, most patients were White, and 39% (n = 263) were obese. More than half of the study population had mild lower urinary tract symptoms (LUTS) before RARP, whereas only a small number had severe LUTS. 

Mean preoperative PSA was 6.9 (SD ± 6.3) ng/mL. National Comprehensive Cancer Network (NCCN) low risk (n = 192; 28%) and intermediate risk (n = 369; 54%) categories comprised the bulk of the patients. Only 2% of the study population received preoperative androgen deprivation therapy (ADT). Of the adverse prostate cancer parameters, 37% had extraprostatic extension, 11% had seminal vesicle invasion, 9% had bladder neck invasion, and 26% had positive surgical margins. Only 11% had a gland that weighed 70 gm or more at pathology after RARP. 

**Figure 2:**
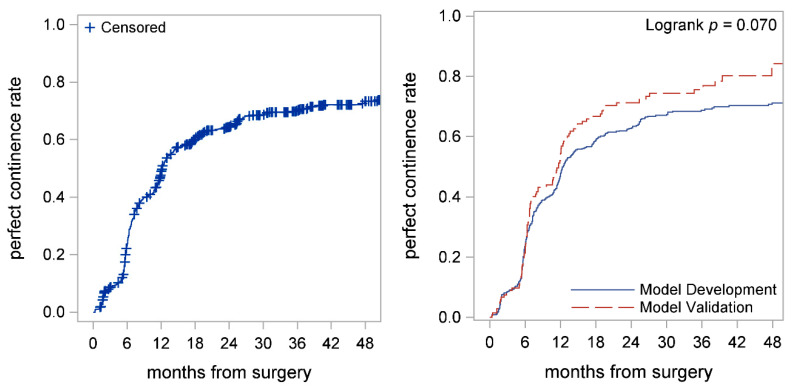
Kaplan–Meier curves showed time to perfect continence for the entire cohort (**left panel**) and time to perfect continence in model development and validation cohorts (**right panel**). Median time to perfect continence was 12.1 (95% CI: 11.7–13.1) months. Perfect continence rates in model development and validation cohorts were similar (*p* = 0.07).

**Figure 3:**
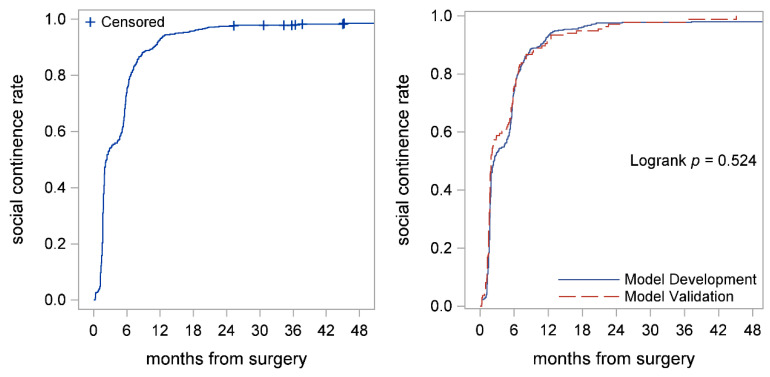
Kaplan–Meier curves showed time to social continence for the entire cohort (**left panel**) and time to social continence in model development and validation cohorts (**right panel**). Median time to social continence was 2.4 (95% CI: 2.0–2.9) months. Social continence rates in model development and validation were similar (*p* = 0.524) (**right panel**).

Overall, median time to perfect continence was 12.1 (95% CI: 11.7–13) months, whereas median time to social continence was 2.4 (95% CI: 2.0–2.9) months (Figure 2 left panel; Figure 3 left panel; and Table 2). The 6-, 12-, and 24- months perfect continence rates were 23% (95% CI: 20–26%), 49% (95% CI: 45–53%), and 64% (95% CI: 61–68%), respectively (Table 2). The 6-, 12-, and 24- months social continence rates were 74% (95% CI: 70–77%), 93% (95% CI: 91–95%), and 97% (95% CI: 96–98%), respectively (Table 2). Time to perfect continence (*p* = 0.070) and social continence (*p* = 0.524) among the model development and model validation cohorts were similar (Figure 2 right panel; Figure 3 right panel).

Univariate predictors for incontinence versus social continence at 6, 12, and 24 months were listed in Supplementary Section Tables S1–S3, respectively. Of the variables evaluated at 6 months, age (both as continuous and dichotomous), BMI (as continuous), and preoperative erectile function (as dichotomous) were found to be significant predictors that differentiated incontinence versus social continence at alpha exit, *p* = 0.1 (Table S1). Similarly, of the variables evaluated at 12 months, only preoperative erectile function (as dichotomous) was found to be a significant predictor that differentiated incontinence versus social continence at alpha exit, *p* = 0.1 (Table S2). At 24-months, BMI (both as continuous and dichotomous) and preoperative erectile function (as dichotomous) were found to be significant predictors that differentiated incontinence versus social continence at alpha exit, *p* = 0.1 (Table S3). 

On multivariate analysis, factors that remained significant predictors that differentiated incontinence versus social continence at 6, 12, and 24 months were listed in Tables S4–S6, respectively. Overall, the main effects selected for the 6-month prediction model were age, race, and preoperative erectile function. The main effect selected for the 12-month model was preoperative erectile function. The main effects selected for the 24-month model were BMI and preoperative erectile function. In order to maintain consistency among the models, the final main effects selected were age, race, BMI, and preoperative erectile function. No interaction terms were included in the model. 

**Figure 4:**
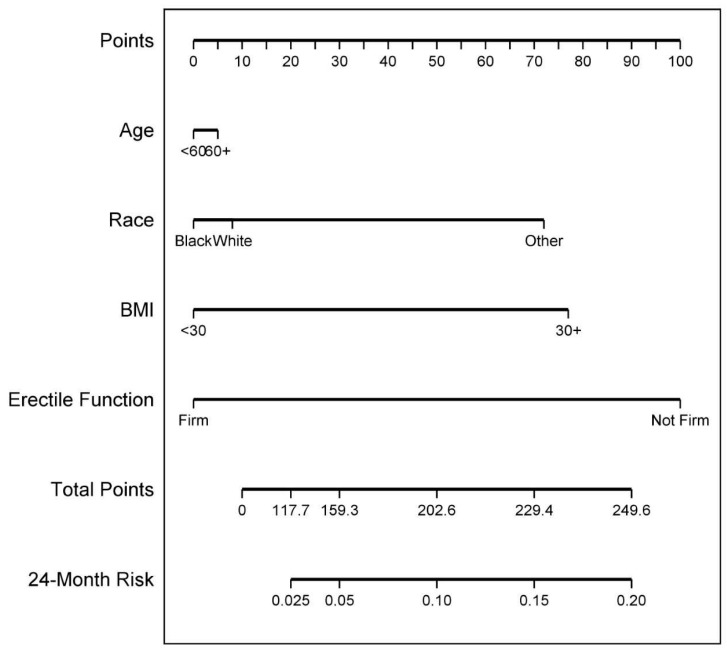
Twenty-four-month incontinence nomogram.

**Figure 5:**
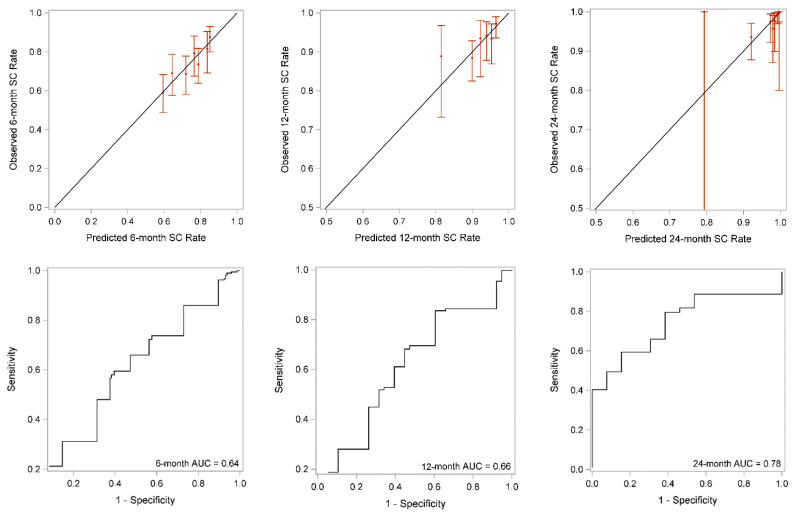
Calibration plots (**upper panels**) and receiver operating curves (ROC) (**lower panels**) that showed initial model performance at 6, 12, and 24 months. SC = social continence; AUC = area under the curve.

**Figure 6:**
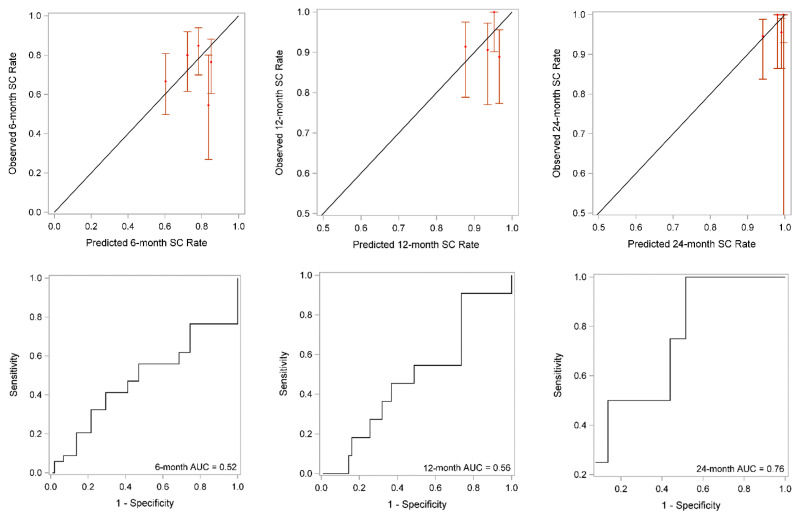
Calibration plots (**upper panels**) and receiver operating curves (ROC) (**lower panels**) that showed validation cohort performance at 6, 12, and 24 months. SC = social continence; AUC = area under the curve.

The 6-, 12-, and 24-month incontinence nomograms were shown in Figures S1, S2, and Figure 4, respectively. The performance of the 6-, 12-, and 24- month models using calibration plots and ROC curves showed weak, and moderate performance with AUCs 0.64, 0.66, and 0.80, respectively (Figure 5). However, the 24-month model tended to over-predict continence at times (Figure 5 upper right panel). When the final model was applied to the validation cohort, the 6-, 12-, and 24-month models performed less well. The 24-month model still exhibited moderate performance but slightly over-estimated continence rates (Figure 6 upper right panel). The 6-, 12-, and 24-month AUCs were 0.52, 0.56, and 0.76, respectively (Figure 6).

## 4. Discussion

A wealth of literature supports the notion that algorithms that incorporate multiple predictive elements, such as nomograms and artificial neural networks, outperform risk assessment based on expert opinion or simpler models [27,28,29]. The development and use of integrated prediction tools can guide counseling and follow-up for men with localized prostate cancer and more accurately identify patients likely to benefit from specific interventions. Sophisticated oncologic nomograms exist that predict prostate cancer-specific survival. To our knowledge, a limited number of predictive tools are available that assess functional outcomes after RARP. Development and utilization of a post-RARP incontinence nomogram could guide counseling, assist with decision-making, and tailor follow-up of men. 

For instance, if an eligible man is faced with a difficult decision of operation versus radiation therapy for treatment of localized prostate cancer, the use of an incontinence after RARP nomogram could prove helpful. Since both modalities offer similar 10- to 15-year cancer-specific survival, the treatment decision may focus on minimizing long term side effects of each treatment option. Therefore, if a man is at high risk for incontinence after RARP based on the incontinence nomogram, he may be counseled more accurately and make a wiser treatment choice.

To our knowledge, only four post-prostatectomy continence recovery nomograms have been published [15,17,30,31]. However, all four of the nomograms have variables that may not be known preoperatively to the urologist or to the patient that involve surgical technique [17,30,31], imaging to measure urethral length [15], prostate volume [17], or surgeon volume [17]. These nomograms may be less patient-specific, more technique-dependent, and less generalizable. The present study developed a reasonably accurate nomogram that always uses available patient-specific preoperative variables to predict incontinence at 6, 12, and 24 months after RARP. 

A priori selection of candidate predictor variables for the development of the incontinence nomogram was based on both clinical utility and statistical significance since incontinence after RARP is multifactorial and multidimensional. From the review of the available literature, the timing and recovery of continence after RARP is influenced by the disturbed anatomy of dissection and patient’s ability to heal [5]. Older men, with significant vascular disease, high BMI, and preoperative erectile dysfunction (used as a surrogate for microvascular disease) take longer to recover continence, if at all [5]. 

More than a decade ago, Lepor et al. demonstrated that the majority of men could take up to 24 months to recover continence in open retropubic radical prostatectomy (RRP) [32]. Eastham et al. reported on a cohort of 581 patients who underwent RRP between 1983 to 1994, and 50% of men were over the age of 63. Age was a significant predictor of early urinary continence recovery on multivariate analysis; over 95% of patients were perfectly continent (defined as 0 or 1 pad for security a day) at 12 months after RRP who were younger than 60 years old [12]. More recent studies of RRP or RARP showed that younger age is a good predictor of early return to continence [6,7,8,9,10,11,12]. Mendiola et al. used a cohort of 300 patients with >1 year follow up, and continence was assessed using validated questionnaires at 1, 3, 6, and 12 months after the operation. They demonstrated that younger men achieved continence (defined as 0 to 1 pad for security daily) sooner than older men when age groups were compared using a 60-year-old cut point (*p* = 0.02) [33]. However, continence outcomes equalized between age groups after 1 year of follow up [33]. Stanford et al. reported data from the Prostate Cancer Outcomes Study, a population-based prospective cohort study of more than 1200 men treated with RRP. They showed that age was associated with continence rates; patients younger than 60 years had a higher chance of continence [34].

Laviguer-Blouin et al. used a 327 patient cohort of men who underwent RARP to demonstrate that IPSS scores were independent predictors of early (within 1 month) return to continence [35]. Jeong et al. observed that prostatic apex shape and membranous urethral length were factors associated with continence recovery at 1 year [24]. Galfano et al. found that a surgical technique sparing the Retzius structures yielded higher early continence rates [26]. Checcucci et al. performed a systematic review and pooled analysis and reported on the importance of a total anatomical reconstruction technique, which resulted in quicker and more frequent continence recovery compared to standard technique [36]. Yet others believe that it is the combination of preservation of bladder neck, pubo-prostatic ligaments, endopelvic fascia, and vesico-urethral complex that is necessary for improved continence recovery [37].

Collette et al. [17] described a model predicting urinary continence at one year after RARP that used age, BMI, IPSS, degree of nerve sparing, prostate volume, ASA score, and surgical caseload as predictors. Matsushita et al. [15] incorporated urethral length, in addition to age, BMI, and ASA score. Tutolo et al. [16] used both preoperative (age, risk stratification, prior androgen deprivation therapy/radiation therapy, urethral stricture disease, stones, recurrent UTIs, etc.) and 3-month post-operative data (intraoperative bleeding, bladder injury, nerve injury/neuropraxia, prolonged lymphorrhea, etc.) to predict continence at 1 year. Finally, Kim et al. [38] confirmed the importance of bilateral neurovascular bundle preservation for early (6 months) recovery of continence after operation.

Like prior studies, our study found that age was a strong predictor for urinary continence recovery at 6 months when treated as either a continuous or dichotomous variable on univariate analysis. Age remained a significant predictor on multivariate analysis. However, age was no longer a significant predictor for differentiating incontinence versus social continence at 24 months, which confirmed an earlier report [33]. Both BMI and preoperative erectile function remained significant predictors of continence recovery in both the 6 and 24 month models. Better erectile function preoperatively may indicate better pelvic vascular supply that allows better healing of the urethrovesical anastomosis [5]. Prostate volume, preoperative LUTS, margin status, bladder neck invasion, seminal vesicle invasion, or a combination of adverse pathologic findings did not contribute significantly to incontinence and therefore were not used for nomogram model development. Finally, race was associated with recovery of urinary control after RARP and was part of all 3 nomograms. Only 16 men identified as a race other than White/Black, most of whom were Asian. Those who identified as another race received up to 100 points on the 6, 12, and 24 months nomograms. This surprising finding was observed also by Basourakos et al. length in a matched cohort study of 36 Asians and 36 non-Asians 3, 6, and 12 months after RARP [39]. A possible explanation was shorter membranous urethral lengths on preoperative MRI. In similar fashion, Black vs. White race entered each nomogram but the racial differences differed by time. Blacks, compared to Whites, suffered a slower recovery of continence at 6 and 12 months, but the racial difference narrowed and favored Blacks at 24 months. These findings in our cohort of 54 Blacks at 12 months were similar to those reported in 140 Blacks by DeCastro et al. at 12 months [40]. Perhaps longer follow-up of their cohort would have disclosed better continence recovery 24 months after RARP in Blacks than Whites. The relationship with race appears complex; these small studies are hypothesis-generating and warrant additional study. 

There are several limitations to our study. Despite the large overall cohort, this was a single center, retrospectively analyzed study with a relatively small validation cohort. Each of the five surgeons differed in nerve-sparing, bladder neck preservation, and reconstruction techniques that resulted in numbers of patients too small in various subsets to compare surgeons or operative techniques. Co-morbidities were assessed prospectively, but preoperative neurologic conditions that may affect post-operative continence were not queried and baseline urodynamic study were not performed to assess for detrusor overactivity preoperatively. Data were not collected on the consistency of preoperative pelvic floor exercises and training, which may play a role in recovery of post-operative continence. The degree of urinary incontinence was assessed using a validated questionnaire, but pad size or weight was not measured and patients purchased and used different pads during the study period. A multicenter external validation study will be needed to confirm and generalize the findings. 

The strengths of the study include the use of a large cohort to develop a complex nomogram that uses commonly elicited preoperative risk factors. The technique for RARP varied among the 5 surgeons, which made this study less technique-dependent and more generalizable. The improvement in continence recovery and usefulness of the nomograms with time were expected since tissue healing and bladder and sphincter function improve from 6 to 12 to 24 months after RARP [30].

## 5. Conclusions

A nomogram for incontinence after RARP is a useful prediction and counseling tool. A 6-month and 12-month nomogram, and reasonably strong 24-month nomogram, were devised, and validated as superior to any single clinical variable for predicting risk of incontinence after RARP. The clinical utility of these nomograms in patients contemplating RARP remains to be determined.

## Figures and Tables

**Table 1:** Baseline Patient Characteristics.

Variable	Model Development	Model Validation	Overall
Number of patients	544 (80%)	136 (20%)	680 (100%)
Age (range)	61 (42–79)	60 (45–73)	61 (42–79)
<60	250 (46%)	74 (54%)	324 (48%)
≥60	294 (54%)	62 (46%)	356 (52%)
Race			
White	489 (90%)	121 (89%)	610 (90%)
Black	43 (8%)	11 (8%)	54 (8%)
Other	12 (2%)	4 (3%)	16 (2%)
BMI	29.5 (±4.7)	29.1 (±5.5)	29.4 (±4.8)
<30	324 (60%)	93 (68%)	417 (61%)
≥30	220 (40%)	43 (32%)	263 (39%)
Severity of LUTS			
Mild (IPSS 0–7)	303 (56%)	63 (46%)	366 (54%)
Moderate (IPSS 8–19)	193 (35%)	66 (49%)	259 (38%)
Severe (IPSS 20–35)	48 (9%)	7 (5%)	55 (8%)
Charlson Comorbidity Index			
≤1	477 (88%)	121 (89%)	598 (88%)
≥2	67 (12%)	15 (11%)	82 (12%)
Preoperative PSA (ng/mL)	7.0 (±6.6)	6.6 (±5.3)	6.9 (±6.3)
PSA			
<10 ng/mL	466 (86%)	119 (88%)	585 (86%)
10–20 ng/mL	57 (11%)	12 (9%)	69 (10%)
>20 ng/mL	21 (4%)	5 (4%)	26 (4%)
Clinical T			
T1	345 (63%)	101 (74%)	446 (65%)
T2	186 (34%)	31 (23%)	217 (32%)
T3	12 (2%)	3 (2%)	15 (2%)
Tx	1 (1%)	1 (1%)	2 (1%)
Clinical N			
N0	137 (25%)	21 (15%)	158 (23%)
N1	1 (1%)	0 (0%)	1 (1%)
Nx	406 (74%)	115 (85%)	521 (76%)
Gleason Score			
≤6	116 (21%)	33 (24%)	149 (22%)
7	378 (70%)	98 (72%)	476 (70%)
8–10	50 (9%)	5 (4%)	55 (8%)
NCCN Risk Category			
Low	149 (27%)	43 (32%)	192 (28%)
Intermediate	295 (54%)	74 (54%)	369 (54%)
High	100 (18%)	19 (14%)	119 (18%)
Neoadjuvant ADT			
Yes	13 (2%)	2 (2%)	15 (2%)
No	530 (98%)	134 (98%)	664 (98%)
Extracapsular Extension			
Yes	197 (36%)	53 (39%)	250 (37%)
No	347 (64%)	83 (61%)	430 (63%)
Seminal Vesicle Invasion			
Yes	60 (11%)	11 (8%)	71 (10%)
No	484 (89%)	125 (92%)	609 (90%)
Bladder Neck Invasion			
Yes	47 (9%)	12 (9%)	59 (9%)
No	595 (91%)	124 (91%)	621 (91%)
Surgical Margins Status			
Positive	137 (25%)	40 (29%)	177 (26%)
Negative	407 (75%)	96 (71%)	503 (74%)
Combination of Adverse Pathologic Factors			
0	273 (50%)	66 (49%)	339 (50%)
1	148 (27%)	36 (26%)	184 (27%)
2	73 (13%)	24 (18%)	97 (14%)
3	41 (8%)	8 (6%)	49 (7%)
4	9 (2%)	2 (1%)	11 (2%)
Prostate Volume			
<70 gm	485 (89%)	119 (88%)	604 (89%)
≥70 gm	59 (11%)	17 (12%)	76 (11%)
Preoperative Erectile Function			
Firm enough for penetration	308 (57%)	82 (60%)	390 (57%)
Not firm enough for penetration	236 (43%)	54 (40%)	290 (43%)

Age was reported in years; Percentages were rounded to the nearest integer; BMI = body mass index; LUTS = lower urinary tract symptoms; ±SD = standard deviation; PSA = prostate-specific antigen; NCCN = National Comprehensive Cancer Network; ADT = androgen deprivation therapy.

**Table 2:** Rate and Time to Continence.

Cohort	6-Month Continence Rate (95% CI)	12-Month Continence Rate (95% CI)	24-Month Continence Rate (95% CI)	Median Time to Continence (95% CI)
	Perfect Continence			
Overall	23% (20–26)	49% (45–53)	64% (61–68)	12.1 (11.7–13)
Model Development	23% (20–27)	48% (44–52)	63% (58–67)	12.5 (11.8–14)
Model Validation	21% (15–29)	54% (45–62)	71% (52–68)	11.6 (8.0–12.5)
	Social Continence			
Overall	74% (70–77)	93% (91–95)	97% (96–98)	2.4 (2.0–2.9)
Model Development	74% (70–77)	93% (91–95)	98% (96–99)	2.5 (2.0–3.3)
Model Validation	75% (67–82)	92% (87–96)	97% (93–99)	1.9 (1.8–2.9)

Time reported in months; CI = confidence interval.

## Data Availability

The models used to generate the nomograms and the de-identified data set that was used to generate the nomograms could be made available through collaboration.

## Supplementary Materials

The following are available online at https://www.mdpi.com/article/10.3390/cancers14071644/s1, Figure S1: Six-month incontinence nomogram, Figure S2: Twelve-month incontinence nomogram, Table S1: Univariate Predictors of Incontinence versus Social Continence at 6 Months, Table S2: Univariate Predictors of Incontinence versus Social Continence at 12-Months, Table S3: Univariate Predictors of Incontinence versus Social Continence at 24 Months, Table S4: Multivariate Predictors of Incontinence at 6 Months, Table S5: Multivariate Predictors of Incontinence at 12 Months, and Table S6: Multivariate Predictors of Incontinence at 24 Months.
