# Supplementary material for: Prediction of Incontinence after Robot-Assisted Radical Prostatectomy: Development and Validation of a 24-Month Incontinence Nomogram
Source: Cancers (Basel). 2022 Mar 24;14(7):1644. doi: 10.3390/cancers14071644 (PMC8997126; doi:10.3390/cancers14071644)
Supplement: Supplementary file 1 [file cancers-14-01644-s001.zip › cancers-1602455-supplementary.pdf]

cancers MDPI

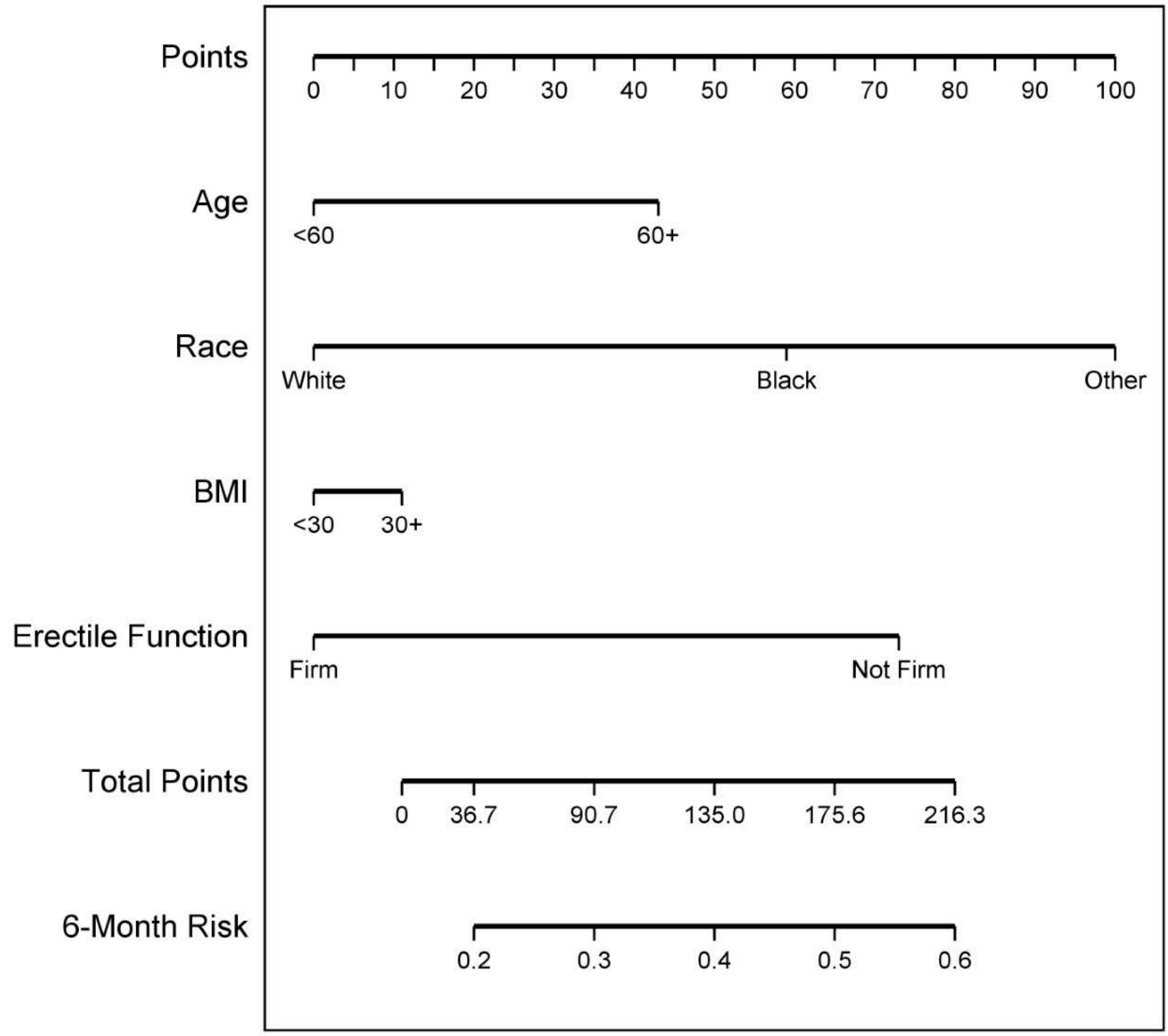

Figure S1: Six-month incontinence nomogram.

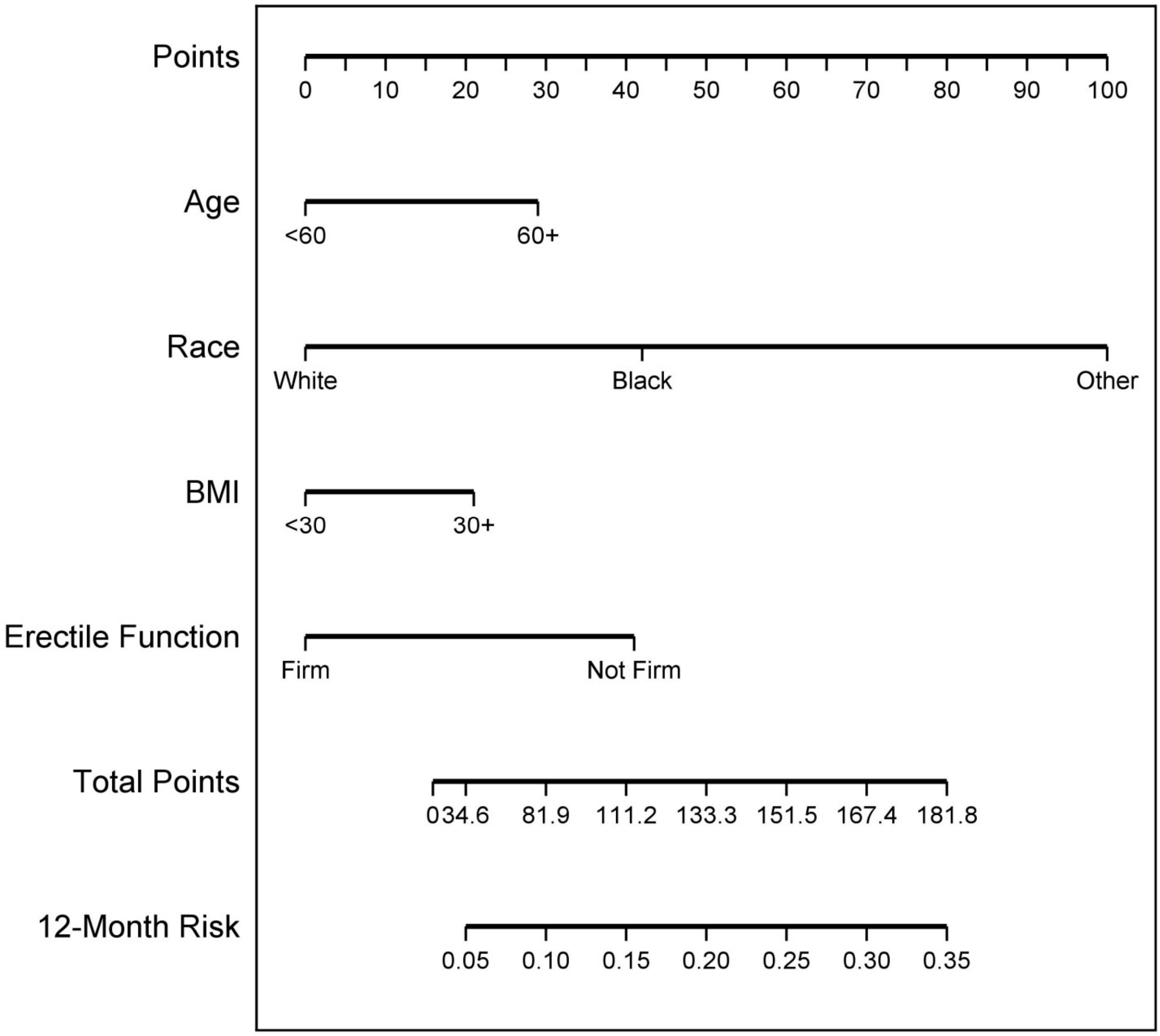

Figure S2: Twelve-month incontinence nomogram.

Table S1: Univariate Predictors of Incontinence versus Social Continence at 6-Months				
	**Incontinence**	**Social Continence**	**OR (95% CI)**	**p-value**
Number of patients	144 (26%)	400 (74%)		
Age (±SD)	62(±6.6)	61(±6.4)	0.97 (0.94-1.00)	0.032
< 60	53 (21%)	197 (79%)	1.00	0.011
≥ 60	91 (31%)	203 (69%)	0.60 (0.41-0.89)	
Race:				
-White	124 (25%)	365 (75%)	1.00	0.198
-Black	16 (37%)	27 (63%)	0.57 (0.30-1.09)	
-Other	4 (44%)	5 (56%)	0.42 (0.11-1.57)	
BMI	30.2 (±5.2)	29.3 (±4.4)	0.96 (0.92-1.00)	0.046
<30	80 (25%)	244 (75%)	1.00	0.253
≥30	64 (29%)	156 (71%)	0.80 (0.54-1.17)	
Severity of LUTS				
Mild (IPSS 0-7)	70 (23%)	233 (77%)	1.00	0.133
Moderate (IPSS 8-19)	59 (31%)	134 (69%)	0.68 (0.45-1.02)	
Severe (IPSS 20-35)	15 (31%)	33 (69%)	0.65 (0.34-1.27)	
Charlson Comorbidity Index				
≤1	126 (26%)	351 (74%)	1.00	0.898
≥2	18 (27%)	49 (73%)	0.96 (0.54-1.71)	
Preoperative PSA (ng/ml)	7.4 (±7.7)	6.8 (±6.1)	0.99 (0.96-1.01)	0.362
PSA				
<10 ng/ml	119 (25%)	347 (75%)	1.00	0.414
10-20 ng/ml	19 (33%)	38 (67%)	0.68 (0.38-1.22)	
>20 ng/ml	6 (29%)	15 (71%)	0.82 (0.31-2.14)	
Clinical T				
-T1	89 (26%)	256 (74%)	1.00	0.848
-T2	53 (26%)	133 (72%)	0.87 (0.58-1.30)	
-T3	2 (17%)	10 (83%)	1.47 (0.34-6.28)	
-Tx	0 (0%)	1 (100%)	1.07 (0.01-102)	
Clinical N				
-N0	41 (30%)	96 (70%)	1.00	0.568
-N1	0 (0%)	1 (100%)	1.31 (0.01-125)	
-Nx	103 (25%)	303 (75%)	1.26 (0.82-1.93)	
NCCN Risk Category				
Low	34 (23%)	115 (77%)	1.00	0.506
Intermediate	82 (28%)	213 (72%)	0.77 (0.49-1.22)	
High	28 (28%)	72 (72%)	0.76 (0.43-1.36)	
Neoadjuvant ADT				
Yes	4 (31%)	9 (69%)	0.76 (0.23-2.47)	0.648
No	140 (26%)	390 (74%)	1.00	

Extracapsular Extension				
Yes	57 (29%)	140 (71%)	0.80 (0.54-1.19)	0.505
No	83 (25%)	254 (75%)	1.00	
Seminal Vesicle Invasion				
Yes	17 (28.3%)	43 (71.7%)	0.89 (0.49-1.61)	0.692
No	127 (26%)	357 (74%)	1.00	
Bladder Neck Invasion				
Yes	13 (28%)	34 (72%)	0.89 (0.46-1.74)	0.525
No	120 (26%)	345 (74%)	1.00	
Surgical Margins Status				
Positive	31 (23%)	106 (77%)	1.30 (0.83-2.05)	0.253
Negative	113 (28%)	294 (72%)	1.00	
Combination of Adverse Pathologic Factors				
0	69 (25%)	204 (75%)	1.00	0.841
1	43 (29%)	105 (71%)	0.82 (0.53-1.29)	
2	20 (27%)	53 (73%)	0.89 (0.50-1.59)	
3	9 (22%)	32 (78%)	1.16 (0.53-2.54)	
4	3 (33%)	6 (67%)	0.63 (0.16-2.55)	
Prostate Volume				
<70 gm	125 (26%)	360 (74%)	1.00	0.275
≥70 gm	19 (32%)	40 (68%)	0.72 (0.40-1.29)	
Preoperative Erectile Function				
Firm enough for penetration	61 (20%)	247 (80%)	2.19 (1.49-3.22)	<.001
Not firm enough for penetration	83 (35%)	153 (65%)	1.00	

Age was reported in years; Percentages were rounded to the nearest integer; BMI = body mass index; LUTS = lower urinary tract symptoms; ±SD = standard deviation; PSA = prostate-specific antigen; NCCN = National Comprehensive Cancer Network; ADT = androgen deprivation therapy.

Table S2: Univariate Predictors of Incontinence versus Social Continence at 12-Months

	**Incontinence**	**Social Continence**	**OR (95% CI)**	**p-value**
Number of patients	38 (7%)	506 (93%)		
Age (±SD)	62(±7.0)	61(±6.4)	0.97 (0.92-1.00)	0.27
< 60	13 (5%)	237 (95%)	1.00	0.14
≥ 60	25 (9%)	269 (91%)	0.60 (0.30-1.20)	
Race:				
-White	31 (6%)	458 (94%)	1.00	0.12
-Black	5 (12%)	38 (88%)	0.48 (0.18-1.27)	
-Other	2 (22%)	10 (78%)	0.21 (0.04-0.97)	
BMI	30.3 (±5.0)	29.5 (±4.6)	0.96 (0.90-1.00)	0.24
<30	19 (6%)	305 (94%)	1.00	0.21

≥30	19 (9%)	201 (91%)	0.66 (0.34-1.27)	
Severity of LUTS				
Mild (IPSS 0-7)	17 (6%)	286 (94%)	1.00	0.07
Moderate (IPSS 8-19)	14 (7%)	179 (93%)	0.76 (0.37-1.56)	
Severe (IPSS 20-35)	7 (15%)	41 (85%)	0.34 (0.13-0.85)	
Charlson Comorbidity Index				
≤1	33 (7%)	444 (93%)	1.00	0.75
≥2	5 (8%)	62 (92%)	0.86 (0.33-2.21)	
Preoperative PSA (ng/ml)	6.7 (±4.8)	7.0 (±6.7)	1.00 (0.95-1.04)	0.83
PSA				
<10 ng/ml	33 (7%)	433 (93%)	1.00	0.72
10-20 ng/ml	3 (5%)	54 (95%)	1.20 (0.38-3.78)	
>20 ng/ml	2 (10%)	19 (90%)	0.60 (0.15-2.43)	
Clinical T				
-T1	21 (6%)	324 (94%)	1.00	0.58
-T2	16 (9%)	170 (91%)	0.68 (0.35-1.34)	
-T3	1 (8%)	11 (83%)	0.51 (0.08-3.15)	
-Tx	0 (0%)	1 (100%)	0.19 (0.01-18.0)	
Clinical N				
-N0	14 (10%)	123 (90%)	1.00	0.18
-N1	0 (0%)	1 (100%)	0.35 (0.01-32.4)	
-Nx	24 (6%)	382 (94%)	1.83 (0.93-3.63)	
NCCN Risk Category				
Low	7 (5%)	142 (95%)	1.00	0.46
Intermediate	24 (8%)	271 (92%)	0.58 (0.25-1.36)	
High	7 (7%)	93 (93%)	0.66 (0.23-1.87)	
Neoadjuvant ADT				
Yes	1 (8%)	12 (92%)	0.63 (0.11-1.36)	0.62
No	37 (7%)	493 (93%)	1.00	
Extracapsular Extension				
Yes	16 (8%)	181 (92%)	0.71 (0.36-1.39)	0.15
No	20 (6%)	317 (94%)	1.00	
Seminal Vesicle Invasion				
Yes	4 (7%)	56 (93%)	0.96 (0.34-2.69)	0.94
No	34 (7%)	450 (93%)	1.00	
Bladder Neck Invasion				
Yes	0 (0%)	47 (100%)	8.07 (0.47-138)	0.35
No	36 (8%)	429 (92%)	1.00	
Surgical Margins Status				
Positive	8 (6%)	129 (94%)	1.23 (0.56-2.71)	0.61
Negative	30 (7%)	377 (93%)	1.00	

Combination of Adverse Pathologic Factors				
0	18 (7%)	255 (75%)	1.00	0.94
1	12 (8%)	136 (71%)	0.79 (0.37-1.67)	
2	6 (8%)	67 (73%)	0.75 (0.29-1.92)	
3	2 (5%)	39 (78%)	1.14 (0.29-4.54)	
4	0 (0%)	6 (67%)	1.38 (0.07-28.6)	
Prostate Volume				
<70 gm	31 (7%)	454 (93%)	1.00	0.10
≥70 gm	7 (12%)	52 (88%)	0.49 (0.21-1.14)	
Preoperative Erectile Function				
Firm enough for penetration	15 (5%)	293 (95%)	2.08 (1.07-4.06)	0.03
Not firm enough for penetration	23 (10%)	213 (90%)	1.00	

Age was reported in years; Percentages were rounded to the nearest integer; BMI = body mass index; LUTS = lower urinary tract symptoms; SD = standard deviation; PSA = prostate-specific antigen; NCCN = National Comprehensive Cancer Network; ADT = androgen deprivation therapy.

**Table S3:** Univariate Predictors of Incontinence versus Social Continence at 24-Months				
	**Incontinence**	**Social Continence**	**OR (95% CI)**	**p-value**
Number of patients	13 (2%)	531 (98%)		
Age (±SD)	63(±8.8)	61(±6.4)	0.96 (0.88-1.04)	0.334
< 60	5 (2%)	245 (98%)	1.00	0.613
≥ 60	8 (3%)	286 (97%)	0.76 (0.25-2.24)	
Race:				
-White	12 (3%)	477 (98%)	1.00	0.761
-Black	1 (2%)	42 (98%)	0.74 (0.13-4.23)	
-Other	0 (0%)	9 (100%)	0.50 (0.02-10.5)	
BMI	32.8 (±5.9)	29.4 (±4.6)	0.89 (0.81-0.97)	0.009
<30	3 (1%)	321 (99%)	1.00	0.015
≥30	10 (5%)	210 (95%)	0.22 (0.06-0.74)	
Severity of LUTS				
Mild (IPSS 0-7)	6 (2%)	297 (98%)	1.00	0.706
Moderate (IPSS 8-19)	6 (3%)	187 (97%)	0.63 (0.21-1.90)	
Severe (IPSS 20-35)	1 (2%)	47 (98%)	0.69 (0.11-4.26)	
Charlson Comorbidity Index				
≤1	11 (2%)	466 (98%)	1.00	0.541
≥2	2 (3%)	65 (97%)	0.65 (0.16-2.62)	
Preoperative PSA (ng/ml)	6.93 (±4.0)	7.00 (±6.6)	0.98 (0.93-1.03)	0.357
PSA				
<10 ng/ml	11 (2%)	455 (98%)	1.00	0.717
10-20 ng/ml	2 (3%)	55 (97%)	0.56 (0.14-2.29)	
>20 ng/ml	0 (0%)	21 (100%)	1.09 (0.06-20.3)	
Clinical T				
-T1	8 (2%)	337 (98%)	1.00	0.720
-T2	5 (3%)	181 (97%)	0.83 (0.28-2.5)	
-T3	0 (0%)	12 (100%)	0.63 (0.03-12.9)	
-Tx	0 (0%)	1 (100%)	0.08 (0.00-7.3)	
Clinical N				
-N0	4 (3%)	133 (97%)	1.00	0.461
-N1	0 (0%)	1 (100%)	0.10 (0.00-10.0)	
-Nx	9 (2%)	397 (98%)	1.41 (0.45-4.42)	
NCCN Risk Category				
Low	3 (2%)	146 (98%)	1.00	0.848
Intermediate	7 (2%)	288 (98%)	0.92 (0.25-3.33)	
High	3 (3%)	97 (97%)	0.67 (0.15-3.01)	
Neoadjuvant ADT				
Yes	0 (0%)	13 (100%)	0.70 (0.04-13.8)	0.817
No	13 (4%)	517 (96%)	1.00	

Extracapsular Extension				
Yes	7 (4%)	190 (96%)	0.50 (0.17-1.45)	0.412
No	6 (2%)	331 (98%)	1.00	
Seminal Vesicle Invasion				
Yes	2 (3%)	58 (97%)	0.57 (0.14-2.31)	0.430
No	11 (2%)	473 (98%)	1.00	
Bladder Neck Invasion				
Yes	0 (0%)	47 (100%)	2.83 (0.16-49.9)	0.709
No	13 (2.8%)	452 (97.2%)	1.00	
Surgical Margins Status				
Positive	3 (2%)	134 (98%)	1.02 (0.30-3.47)	0.981
Negative	10 (3%)	397 (97%)	1.00	
Combination of Adverse Pathologic Factors				
0	6 (2%)	267 (98%)	1.00	0.831
1	3 (2%)	145 (98%)	1.01 (0.27-3.78)	
2	3 (4%)	70 (96%)	0.49 (0.13-1.86)	
3	1 (2%)	40 (98%)	0.66 (0.11-4.07)	
4	0 (0%)	9 (100%)	0.46 (0.02-10.2)	
Prostate Volume				
<70 gm	11 (2%)	474 (98%)	1.00	0.415
≥70 gm	2 (3%)	57 (97%)	0.56 (0.14-2.27)	
Preoperative Erectile Function				
Firm enough for penetration	11 (5%)	225 (95%)	1.00	0.009
Not firm enough for penetration	2 (1%)	306 (99%)	6.25 (1.57-24.9)	

Age was reported in years; Percentages were rounded to the nearest integer; BMI = body mass index; LUTS = lower urinary tract symptoms; SD = standard deviation; PSA = prostate-specific antigen; NCCN = National Comprehensive Cancer Network; ADT = androgen deprivation therapy.

Table S4: Multivariate Predictors of Incontinence at 6-Months

	**Variable**	**OR (95% CI)**	**p-value**
Age	≥60 vs <60	0.66 (0.44, 0.99)	0.04
Race	Black vs White	0.57 (0.29, 1.11)	0.20
	Other vs White	0.37 (0.03, 5.08)	
BMI	≥30 vs <30	0.90 (0.60, 1.35)	0.61
Pre-OP Erectile Function	Firm Enough vs Not Firm Enough	2.02 (1.34, 3.04)	<.001

Firm enough for penetration; Not firm enough for penetration; BMI = body mass index.

**Table S5:** Multivariate Predictors of Incontinence at 12-Months			
	**Variable**	**OR (95% CI)**	**p-value**
Age	≥60 vs <60	0.64 (0.32, 1.31)	0.22
Race	Black vs White	0.52 (0.18, 1.51)	0.25
	Other vs White	0.20 (0.01, 3.07)	
BMI	≥30 vs <30	0.73 (0.36, 1.45)	0.37
Pre-OP Erectile Function	Firm Enough vs Not Firm Enough	1.90 (0.92, 3.91)	0.08

Firm enough for penetration; Not firm enough for penetration; BMI = body mass index.

Table S6: Multivariate Predictors of Incontinence at 24-Months

	**Variable**	**OR (95% CI)**	**p-value**
Age	≥60 vs <60	0.93 (0.28, 3.05)	0.90
Race	Black vs White	1.15 (0.26, 5.15)	0.98
	Other vs White	0.33 (0.02, 6.85)	
BMI	≥30 vs <30	0.25 (0.06, 1.02)	0.05
Pre-OP Erectile Function	Firm Enough vs Not Firm Enough	5.71 (1.22, 26.81)	0.03

Firm enough for penetration; Not firm enough for penetration; BMI = body mass index.
